# Operative Therapie von Nävi bei Kindern in einem dermatochirurgischen Zentrum: Histopathologie und Komplikationen

**DOI:** 10.1007/s00105-023-05147-0

**Published:** 2023-04-29

**Authors:** Katrin Kofler, Hans-Martin Häfner, Stephan Forchhammer, Lukas Kofler

**Affiliations:** 1 Universitätshautklinik Tübingen, Liebermeisterstr. 25, 72076 Tübingen, Deutschland; 2 Hautzentrum skin+more MVZ, Holzmarkt 6, 88400 Biberach a.d.R., Deutschland; 3 Zentrum für Seltene Hauterkrankungen/Kongenitale Nävi, Universitätshautklinik Tübingen, Liebermeisterstr. 25, 72076 Tübingen, Deutschland

**Keywords:** Melanozytärer Nävus, Dermatochirurgie, Kongenitaler Nävus, Exzision, Histologische Sicherung, Melanocytic nevus, Dermatologic surgery, Congenital nevus, Excision, Histological confirmation

## Abstract

**Hintergrund:**

Im Kindesalter ist die Indikationsstellung zur operativen Therapie und histologischen Diagnostik bei melanozytären Nävi eine große Herausforderung im klinischen Alltag. In der Beratung der Kinder und Eltern nimmt auf der einen Seite der Ausschluss von malignen Befunden, auf der anderen Seite das Risiko für Komplikationen die größte Rolle ein.

**Patienten und Methodik:**

Es wurden 946 Kinder unter 10 Jahren eingeschlossen, die mit der Verdachtsdiagnose eines Melanozytennävus an der Universitätshautklinik Tübingen zwischen 2008 und 2018 operiert wurden. Erfasst wurden der dermatohistopathologische Befund sowie postoperative Komplikationen.

**Ergebnisse:**

Die klinische Diagnose eines melanozytären Nävus wurde in 93,2 % (882/946) der Fälle histologisch bestätigt. Darunter fanden sich 41 Spitz-Nävi und 18 pigmentierte Spindelzelltumoren. Bei 2 Kindern (0,2 %) aus dem Kollektiv wurde die Diagnose eines Melanoms gestellt. Bei weiteren 6,6 % fanden sich nichtmelanozytäre Befunde (u. a. Naevus sebaceus, epidermale Nävi). Die Komplikationsrate war mit 3 % gering. Die häufigste Komplikation war in 1,7 % das Auftreten eines postoperativen Wundinfektes.

**Schlussfolgerung:**

Es ist auch im Kleinkindesalter möglich, kongenitale Nävi unterschiedlicher Größe zu biopsieren oder operativ zu entfernen. Ein wichtiges Instrument hierfür ist die serielle Exzision kongenitaler Nävi. Im untersuchten Kollektiv zeigte sich eine geringe Komplikationsrate. Eine histologische Sicherung ist bei klinisch suspekten oder untypischen Befunden unabdingbar.

## Abstract (en)

**Background:**

The indication for surgical management and histological diagnosis of melanocytic nevi in children is a major challenge in clinical routine. In consultations with children and parents, the exclusion of malignant findings, on the one hand, and the risk of complications, on the other hand, are important.

**Patients and methodology:**

Included were 946 children under the age of 10 years who underwent surgery with a suspected diagnosis of melanocytic nevus at the University Department of Dermatology, Tübingen, Germany, between 2008 and 2018. Dermatohistopathologic findings and postoperative complications were recorded.

**Results:**

A clinical diagnosis of melanocytic nevus was histologically confirmed in 93.2% (882/946) of cases, whereby there were 41 Spitz nevi and 18 pigmented spindle cell tumors. Melanoma was diagnosed in 2 of the children (0.2%). In another 6.6%, non-melanocytic findings (e.g., nevus sebaceous, epidermal nevi) were diagnosed. The complication rate was low at 3%. The most common complication was the occurrence of postoperative wound infection in 1.7%.

**Conclusion:**

It is possible to take a biopsy or surgically remove congenital nevi of different sizes even in infants. Serial excision of congenital nevi is an important tool for this purpose. In the investigated cohort, the complication rate was low. Histological confirmation is essential in case of clinically suspicious or atypical findings.

Die operative Therapie von Hautveränderungen bei Säuglingen und Kindern ist ein anspruchsvoller Teil innerhalb der Dermatochirurgie. Neben der Wahl des operativen Vorgehens sind v. a. die Indikationsstellung sowie der Zeitpunkt des Eingriffes und die Aufklärung der Eltern hinsichtlich zu erwartender Komplikationen eine große Herausforderung im klinischen Alltag. Das operative Spektrum umfasst hier sowohl kleine Befunde als auch großflächige Nävi sowie Befunde an Problemlokalisationen (Abb. 1). Neben diagnostischen Biopsien ist es auch möglich, bereits im Kleinkindesalter Serienexzisionen zur operativen Therapie von Hautveränderungen wie kongenitalen Nävi, Naevi sebacei oder anderen selteneren kongenitalen Befunden durchzuführen. Exzisionen bei Kindern führen wegen der größeren Hautelastizität und der guten Geweberegeneration zu kosmetisch sehr guten Ergebnissen und ermöglichen eine histologische Diagnosesicherung [6].

**Figure 1:**
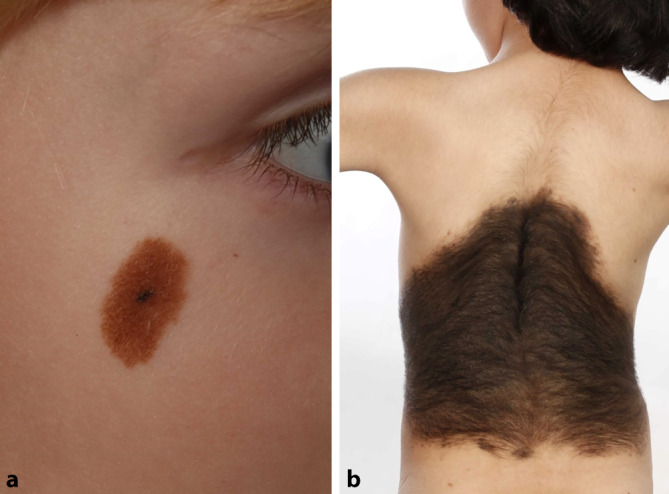

Bei rund 1 % aller lebend geborenen Kinder finden sich kongenitale Nävi [7]. Es muss hier zwischen kongenitalen Nävi und kongenitalen Riesennävi unterschieden werden, die deutlich seltener auftreten. Verlässliche Inzidenzzahlen liegen nicht vor, da in Deutschland weder eine Meldepflicht noch ein vollständiges Register besteht. Von großen kongenitalen Nävi spricht man, wenn im Erwachsenenalter ein Durchmesser von mehr als 20 cm angenommen werden kann, von kongenitalen Riesennävi bei einem angenommenen Durchmesser von mehr als 40 cm. Das Lebenszeitrisiko einer malignen Entartung von kongenitalen Nävi wird in der Literatur kontrovers diskutiert. Für kongenitale Riesennävi besteht das höchste Lebenszeitrisiko und wird zwischen 5 und 15 % geschätzt [1]. Die Inzidenz von pädiatrischen Melanomen lag insgesamt in einem Kollektiv aus den Niederlanden bei 0,67 pro 100.000 pro Jahr [5].

Bei malignitätssuspekten Hautveränderungen im Kindesalter stellt die Exzision oder Biopsie mit anschließender histologischer Aufarbeitung den Goldstandard zur Diagnostik dar. Darüber hinaus besteht eine relative Indikation zur operativen Therapie, wenn funktionelle Einschränkungen (wie rezidivierende Infekte bei verrukösen Anteilen kongenitaler Nävi) oder Hautveränderungen mit Potenzial zur Stigmatisierung (wie große kongenitale Nävi an sichtbaren Körperstellen) vorliegen. Die Indikation zur Exzision sollte gemeinsam mit den Eltern und unter Berücksichtigung aller individuellen Risiken getroffen werden. Insbesondere wenn keine absolute Indikation bei Malignitätsverdacht vorliegt bzw. bei klinisch unauffälligen kleineren Nävi ohne signifikante Entartungstendenz ist eine Exzision nicht zwingend indiziert. Bei kleinen kongenitalen Nävi an wenig sichtbaren Körperstellen und damit geringem Stigmatisierungspotenzial kann die sequenzielle Dermatoskopie eine relevante Alternative sein, insbesondere da es sich um eine nichtinvasive, kostengünstige und komplikationsfreie Technik handelt.

Für die operative Therapie von kongenitalen Nävi wurden verschiedene Techniken beschrieben [8]. Neben plastischen Deckungsverfahren kommt insbesondere der Serienexzision in Powerstretching-Technik eine besondere Rolle zu [3, 6]. Eingriffe können einerseits in Vollnarkose [4], andererseits (in speziellem Setting) auch als serielle Eingriffe in Tumeszenzlokalanästhesie durchgeführt werden [6, 10, 11].

Ziel dieser Studie war es, das Spektrum der dermatohistopathologischen Diagnosen von Hautveränderungen zu untersuchen, die bei Kindern bis zum Alter von 10 Jahren operativ entfernt oder biopsiert wurden. Außerdem sollte evaluiert werden, wie häufig Komplikationen bei Kindern unter 10 Jahren bei dermatologischen operativen Eingriffen in diesem Zusammenhang auftreten.

## Material und Methoden

### Patientenkollektiv

In die retrospektive Analyse wurden alle Kinder, die jünger als 10 Jahre alt waren und über den Zeitraum von 2008 bis 2018 in der Universitätshautklinik Tübingen mit der Verdachtsdiagnose Melanozytennävus (codiert als „D22.x“ nach ICD-10) operiert wurden, eingeschlossen. Insgesamt konnten so 946 Kinder identifiziert und in die Auswertung einbezogen werden. Alle Befunde wurden im Rahmen der Erstvorstellung auch dermatoskopisch evaluiert.

Alle Gewebeproben wurden von erfahrenen Dermatohistopathologen der Universitätshautklinik Tübingen befundet. Alle Proben wurden primär am H&E(Hämatoxylin und Eosin)-gefärbten Schnittpräparat befundet. Bei Notwendigkeit einer weiterführenden immunhistochemischen Aufarbeitung erfolgten individuell je nach Befund Färbungen mit Antikörpern, insbesondere gegen Melan A, Hmb45, S100, Sox10, Ki67, Histon H3 und p16. Bei besonderen Indikationen, insbesondere zur Abgrenzung von atypischen spitzoiden Nävi gegenüber spitzoiden Melanomen sowie zur Abgrenzung proliferierender Knoten gegenüber Melanomen auf dem Boden eines kongenitalen Nävus erfolgten bei Bedarf molekularpathologische Untersuchungen mittels Array-CGH(„array-based comparative genomic hybridization“)-Analyse und NGS(„next generation sequencing“)-Diagnostik.

Die Datenerhebung fand auf Grundlage der vorliegenden dermatohistopathologischen Befunde, der Operationsberichte und der Patientenakte statt. Ausgewertet wurden das Alter bei der ersten Operation, die dermatohistopathologischen bzw. pathologischen Befunde, die Lokalisation der Hautveränderung, Anzahl und Größe der operativen Eingriffe sowie der postoperative Verlauf unter Berücksichtigung von postoperativen Komplikationen.

Die Angaben zu Länge und Breite des jeweils (spindelförmig) resezierten Nävusanteils wurden aus dem Operationsbericht entnommen, und anschließend wurde ein Mittelwert aller exzidierten Längen bzw. Breiten berechnet.

Die Studie wurde von der Ethikkommission an der Medizinischen Fakultät der Eberhard-Karls-Universität und am Universitätsklinikum Tübingen genehmigt (Nummer des Studienprotokolls: 664/2020BO2).

### Operatives Vorgehen

In die vorliegende Untersuchung wurden sowohl Kinder, bei denen eine diagnostische Biopsie durchgeführt wurde, als auch Kinder, die eine oder mehrere Operationen zur Reduktion bzw. Resektion von kongenitalen (Riesen‑)Nävi erhielten. Die Eingriffe wurden sowohl in automatisierter Tumeszenzlokalanästhesie als auch in Vollnarkose durchgeführt. Im Rahmen der automatisierten Tumeszenzlokalanästhesie wird eine hochverdünnte Tumeszenzlösung (bei Kindern 0,05 %) aus Lidocain und Ropivacain sowie einem Adrenalinzusatz mittels einer automatisierten Pumpe (Infusomat P, B. Braun Melsungen, Melsungen, Deutschland) mit vordefinierten Zeitvolumen infundiert.

### Statistische Auswertung

JMP® 11 Software (JMP® Software, Marlow, Buckinghamshire, England) wurde für die Datenanalyse verwendet. Die Daten wurden als Median sowie absolute und relative Häufigkeiten ausgedrückt.

## Ergebnisse

### Patientenkollektiv

Insgesamt konnten 946 Kinder in die Studie eingeschlossen werden. Das Alter der Kinder lag im Median bei 4,4 Jahren (Range: 5 Tage bis 9,98 Jahre).

Von den untersuchten Kindern waren 54 % weiblich (*n* = 509) und 46 % männlich (*n* = 437). Bei den durchgeführten Eingriffen handelte es sich in 91,9 % um Exzisionen und in 8,1 % um Biopsien (Tab. 1).

**Table 1:** 

*Geschlecht*	*n (%)*
Weiblich	509 (54 %)
Männlich	437 (46 %)
*Alter bei Erstoperation*	*Median*
4,4 Jahre	5 Tage bis 9,98 Jahre
*Operation Lokalisation*	*n*
Kopf/Hals	306
Stamm	205
Untere Extremität	185
Obere Extremität	130
Multiple Lokalisationen	120
*Art des Eingriffes*	*n (%)*
Exzision	869 (91,9 %)
Biopsie	77 (8,1 %)
*Gesamtdefektgröße*	*cm (Mittelwert)*
Länge	21,4
Breite	19,1

### Histologische Befunde

Bei der histologischen Aufarbeitung zeigten sich in 93,2 % der Fälle melanozytäre Nävi (882/946). Hierbei handelt es sich in den meisten Fällen um kongenitale melanozytäre Nävi (70,6 %; 668/946) und nichtkongenitale melanozytäre Nävi (14,4 %; 136/946). Bei insgesamt 41 Kindern wurde die Diagnose eines Spitz-Nävus gestellt (4,3 %), bei 18 Kindern die eines pigmentierten Spindelzelltumors (Reed) (1,9 %), bei 11 Kindern die eines blauen Nävus (1,2 %) sowie bei 8 Kindern die eines dysplastischen Nävus (0,8 %). Die Verdachtsdiagnose eines Melanozytennävus (D22.x) wurde somit in 93,2 % der Fälle bestätigt. Bei 0,2 % der Kinder wurde die Diagnose eines Melanoms gestellt (2/946). Es zeigten sich jeweils ein spitzoides Melanom und ein Melanom auf dem Boden eines kongenitalen Nävus.

Klinisch wurden 20 Hautveränderungen als Verdacht auf Spitz-Nävi eingestuft, wobei sich diese Diagnose in 16 Fällen (80 %) bestätigen ließ. Andererseits wurden 25 Hautveränderungen, die histologisch als Spitz-Nävus befundet wurden, klinisch anders eingeschätzt: In 24 % (6/25) war die klinische Verdachtsdiagnose ein kongenitaler Nävus, in 36 % (8/25) ein Nävuszellnävus, in 16 % (4/25) ein dysplastischer Nävus. Jeweils 1‑mal (entsprechend 4 % [1/25]) war die klinische Verdachtsdiagnose eines histologisch gesicherten Nävus Spitz ein Nävus Reed, ein Dermatofibrom, ein Naevus papillomatosus, ein Basalzellkarzinom, ein Lymphozytom bzw. ein Granuloma pyogenicum.

Nichtmelanozytäre Befunde zeigten sich bei 6,6 % (62/946) in der histologischen Aufarbeitung und stimmten somit nicht mit der klinischen Verdachtsdiagnose eines Melanozytennävus überein. Hierbei wurde am häufigsten die Diagnose eines Naevus sebaceus (25/946) oder eines epidermalen Nävus (13/946) gestellt. Weitere, seltenere histologische Diagnosen waren u. a. Hamartome, Hämangiome, Aplasia cutis congenita und ein juveniles Xanthogranulom (Tab. 2).

**Table 2:** 

Histologische Diagnose	n
*Melanozytäre Nävi*	*882*
Kongenitaler melanozytärer Nävus	668
Nichtkongenitaler melanozytärer Nävus	136
Spitz-Nävus	41
Pigmentierter Spindelzelltumor (Reed)	18
Blauer Nävus	11
Dysplastischer melanozytärer Nävus	8
*Melanom*	*2*
Melanom auf kongenitalem Nävus	1
Spitzoides Melanom	1
*Weitere Diagnosen*	*62*
Naevus sebaceus	25
Epidermaler Nävus	13
Hamartom	6
Hämangiom	6
Aplasia cutis congenita	3
Juveniles Xanthogranulom	2
B‑Zell-Pseudolymphom	1
Dermaler Abszess	1
Dermatofibrom	1
Granuloma pyogenicum	1
Neurofibrom	1
Kutane Leishmaniose	1
Lymphangiom	1

### Postoperative Komplikationen

Bei 3 % der Kinder traten postoperative Komplikationen auf (28/946). Hier war die häufigste Komplikation bei 1,7 % (16/946) der Kinder das Auftreten eines Wundinfektes. Eine stationäre Therapie aufgrund eines Wundinfektes war bei keinem dieser Kinder erforderlich. Bei 0,6 % (6/946) kam es zu einer Blutung, bei 0,5 % (5/946) zeigte sich eine Nahtdehiszenz, und bei 0,3 % (3/946) kam es aufgrund von Fadenresiduen zu Beschwerden im postoperativen Verlauf.

Wenn nur Exzisionen von großen Nävi (definiert nach Ruiz-Maldona mit einem Durchmesser von größer als 11 cm) betrachtet wurden, traten Komplikationen in insgesamt 3,1 % (25/810) auf. Dabei fanden sich auch hier am häufigsten Infekte in 1,9 % (15/810), gefolgt von Blutungen mit 0,6 % (5/810), einer Nahtdehiszenz in 0,6 % (5/810) und Fadenresiduen in 0,4 % (3/810).

## Diskussion

Die operative Entfernung von pigmentierten Hautveränderungen und die Biopsie suspekter Befunde stellen nicht nur bei Erwachsenen, sondern auch im Kindesalter eine häufige Indikation für einen dermatochirurgischen Eingriff dar. Hierbei hat die Abgrenzung gutartiger von bösartigen Befunden die höchste Priorität. Die klinische sowie auch histopathologische Differenzierung zwischen Melanomen und spitzoiden melanozytären Nävi ist dabei eine besondere Herausforderung. Einige dieser Tumoren lassen sich allein auf Grundlage der morphologischen und immunhistochemischen Befunde nicht weiter differenzieren, hier sind molekularpathologische Untersuchungen wie die CGH(„comparative genomic hybridization“)-Analyse zur Detektion quantitativer chromosomaler Aberrationen oder die NGS(„next generation sequencing“)-Diagnostik zum Nachweis Spitz-typischer oder Spitz-untypischer Mutationen nötig [2, 9].

**Figure 2:**
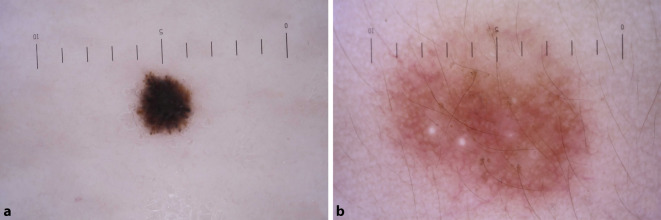

In unserem Kollektiv fanden sich 41 Kinder mit einem Spitz-Nävus und 18 Kinder mit einem pigmentierten Spindelzelltumor (insgesamt 6,2 %) (Abb. 2). Weiter zeigten sich dysplastische (1,2 %) und blaue Nävi (0,8 %), die sich klinisch teilweise schwer von Melanomen abgrenzen lassen. Der pigmentierte Spindelzellnävus Reed sowie der Spitz-Nävus können sich in der klinischen Diagnose als schwierig präsentieren und bedürfen dermatoskopischer Erfahrung, wofür die Breite der klinischen Verdachtsdiagnosen beim Spitz-Nävus spricht. Bei 25 schließlich histologisch als Spitz-Nävi diagnostizierten Läsionen wurde initial eine abweichende klinische Diagnose gestellt. Dies unterstreicht die Bedeutung einer konsequenten Dermatoskopie und histologischen Sicherung bei unklarem Befund. Insgesamt handelt es sich bei Melanomen im Kindesalter um eine sehr seltene Diagnose. Für das Jahr 2013 wurde eine Inzidenz von 0,22 pro 100.000 Kinder unter 11 Jahren in den Niederlanden berechnet [5]. In unserer Kohorte wurde bei 2 Kindern histologisch die Diagnose eines Melanoms gestellt, entsprechend 0,2 % der untersuchten Gewebeproben. Dies unterstreicht die Relevanz der histologischen Aufarbeitung bei klinisch suspekten Befunden auch im Kindesalter.

Bei 6,6 % der Kinder ergaben sich, abweichend von der initialen Verdachtsdiagnose, nichtmelanozytäre Befunde. Im Einzelfall kann es im klinischen Alltag – v. a. bei Befunden an untypischen Lokalisationen oder bei ungewöhnlicher klinischer Präsentation – herausfordernd sein, maligne Befunde rein klinisch auszuschließen. In diesen Situationen kommen der Exzision bzw. Biopsie und histologischen Aufarbeitung zur exakten Diagnosestellung und Festlegung einer weiteren Therapienotwendigkeit große Bedeutung zu.

Die ICD-10-Codierung mit D22.x stellt ein Sammelbecken von unterschiedlichen histologischen Diagnosen benigner melanozytärer Hautveränderungen dar. Eine Unterscheidung zwischen kongenitalen, nichtkongenitalen Nävi, dysplastischen Nävi oder blauen Nävi ist hier nicht möglich und auch nicht vorgesehen. Auch eine Abgrenzung zu den Melanomsimulatoren Spitz-Nävus und pigmentiertem Spindelzelltumor wird nicht vorgenommen. Diese Unschärfe impliziert eine Limitierung der vorliegenden Untersuchung. Allerdings wurde hier bewusst eine Analyse eines „real-world setting“ gewählt, um den klinischen Alltag darzustellen. Es wurden daher neben Exzisionen auch Biopsien in dieser Analyse berücksichtigt, die im Kollektiv mit 8,1 % nur einen geringen Anteil der Eingriffe ausmachten.

Trotz einer teils erheblichen Größe der Defekte (Tab. 1) zeigte sich eine insgesamt geringe Komplikationsrate von 3 %. Hierbei waren im postoperativen Verlauf das Auftreten eines Wundinfektes oder einer Nachblutung die häufigsten Komplikationen (1,7 % bzw. 0,6 %).

Die Universitäts-Hautklinik Tübingen verwendet bei Kindern sowohl für Biopsien als auch Exzisionen zur Lokalanästhesie eine automatisierte Tumeszenzlokalanästhesie, die sowohl Hydrodissektion als auch blutungsarmes Operieren ermöglicht. Darüber hinaus ist es mit dieser Technik in entsprechendem Setting aufgrund der Schmerzarmut bei geringem Fluss möglich, selbst Kleinkinder zu anästhesieren [10]. Eine Tumeszenzlokalanästhesie ist sogar in den ersten Lebensmonaten bei entsprechender Erfahrung und Operationssetting gut durchführbar und hat das Potenzial, die Anzahl an Eingriffen in Vollnarkose zu vermeiden. Die lang anhaltende Analgesie vermindert zudem das Auftreten von postoperativen Schmerzen und verringert den Einsatz weiterer Schmerzmittel [6, 10].

Eine Exzisionsbiopsie stellt im Gegensatz zu einer reinen Biopsie eine bessere Möglichkeit zur Diagnosesicherung und zur histologischen Abgrenzung zwischen gutartigen und bösartigen Befunden im Kindesalter dar. Zu bedenken ist, dass eine klinisch repräsentative Stelle innerhalb eines größeren Nävus nicht zwingend mit einer sicheren histologischen Diagnose korrespondiert.

Weiter ist die (Serien‑)Exzision zur vollständigen Entfernung oder Größenreduktion von kongenitalen Nävi im Kleinkindalter sicher durchführbar und ermöglicht gleichzeitig eine histologische Aufarbeitung bei niedriger Komplikationsrate.

Limitierungen der vorliegenden Arbeit liegen zum einen in der retrospektiven Analyse der Daten. Darüber hinaus zeigt die zugrunde liegende ICD-10-Codierung eine Unschärfe hinsichtlich der darin integrierten Diagnosen. Insbesondere eine detaillierte Unterscheidung histologischer Diagnosen ist a priori durch die Codierung nicht möglich. Das dargestellte Kollektiv ist inhomogen und evaluiert mit diagnostischen Biopsien und Exzisionen unterschiedliche Indikationsstellungen; dies erschwert den konkreten Vergleich beispielsweise bei Komplikationsraten.

## Fazit

An diesem großen Kollektiv konnte gezeigt werden, dass die klinische Diagnose eines melanozytären Nävus in 6,8 % histologisch nicht bestätigt werden konnte und sich hier sowohl Melanome als auch nichtmelanozytäre Tumoren als Diagnosen zeigten. Dies unterstreicht die Bedeutung einer histologischen Aufarbeitung bei unklaren melanozytären Befunden im Kindesalter. Neben der histologischen Sicherung durch rein diagnostische Biopsien ist es auch im Kleinkindesalter und sogar im Alter von wenigen Monaten an spezialisierten Zentren möglich, kongenitale Nävi unterschiedlicher Größe operativ zu entfernen. Ein wichtiges Instrument hierfür ist die serielle Exzision kongenitaler Nävi. Im untersuchten Kollektiv zeigte sich eine mit insgesamt 3 % geringe Komplikationsrate.
